# Management of Severe Rhabdomyolysis and Exercise-Associated Hyponatremia in a Female with Anorexia Nervosa and Excessive Compulsive Exercising

**DOI:** 10.1155/2016/8194160

**Published:** 2016-09-19

**Authors:** Marwan El Ghoch, Simona Calugi, Riccardo Dalle Grave

**Affiliations:** Department of Eating and Weight Disorders, Villa Garda Hospital, Via Monte Baldo 89, Garda, 37016 Verona, Italy

## Abstract

This case report describes the management of a 49-year-old female with restricting-type anorexia nervosa and excessive compulsive exercising associated with rhabdomyolysis, high levels of serum creatine kinase (CK) (3,238 U/L), and marked hyponatremia (Na^+^: 123 mEq/L) in the absence of purging behaviours or psychogenic polydipsia; it is the first case report to describe exercise-associated hyponatremia in a patient with anorexia nervosa. The patient, who presented with a body mass index (BMI) of 13.4 kg/m^2^, was successfully treated by means of an adapted inpatient version of an enhanced form of cognitive behavioural therapy (CBT-E). Within a few days, careful water restriction, solute refeeding, and the specific cognitive behavioural strategies and procedures used to address the patient's excessive compulsive exercising and undereating produced a marked reduction in CK levels, which normalised within one week. Exercise-associated hyponatremia also gradually improved, with serum sodium levels returning to normal within two weeks. The patient thereby avoided severe complications such as cerebral or pulmonary oedema or acute renal failure and was discharged after 20 weeks of treatment with a BMI of 19.0 kg/m^2^ and improved eating disorder psychopathology.

## 1. Introduction 

Rhabdomyolysis, muscle injury associated with increased serum creatine kinase activity (CK), has been widely reported in those who practice sports [1] and may occur in conjunction with exercise-associated hyponatremia [2]. Although the exact mechanism behind the latter is unknown, several risk factors, including exercise duration, female gender, low body weight, and excessive fluid intake, have been identified [2]. An association between rhabdomyolysis and exercise-associated hyponatremia has been confirmed by recent studies [3, 4]. Some authors have hypothesised that one condition may cause the other [4–6], as rhabdomyolysis may bring about exercise-associated hyponatremia through arginine vasopressin secretion [4, 7], stimulated by the increase of interleukin-6 and CK [8, 9], and exercise-associated hyponatremia may cause rhabdomyolysis through changes in intracellular K^+^ and/or Ca^++^ concentrations, which reduce cell membrane stability and cause muscle cell injury [10]. In contrast, others have reported that both conditions may occur independently [4], and this association therefore remains to be proven conclusively. However, it is known that the simultaneous coexistence of rhabdomyolysis and exercise-associated hyponatremia creates a complicated and opposing treatment paradox as regards liquid resuscitation [3, 11], increasing the risk of severe complications, such as acute renal failure and/or cerebral and pulmonary oedema [12, 13].

Excessive exercising is a common behaviour in patients with anorexia nervosa, an eating disorder characterized by significantly low weight, intense fear of weight gain, and a psychopathological disturbance in the way in which one's body weight or shape is experienced [14, 15]. Exercise is defined as “excessive” when it significantly interferes with important activities, occurs at inappropriate times or in inappropriate settings, or continues despite injury or other medical complications [16]. In such patients, generally females, excessive exercising often results in overuse injuries, bone fractures [17], and cardiac complications [18]. It also hinders weight restoration and seems to be a negative predictor of treatment outcome [19]. However, the hyponatremia observed in some patients with anorexia nervosa is commonly associated with the adoption of purging behaviours (i.e., self-induced vomiting and laxative and diuretic misuse) [20] or excessive water intake by psychogenic polydipsia, rather than excessive compulsive exercising [21]. Some reports have also described the occurrence of rhabdomyolysis in patients with anorexia nervosa [22–25], but indications as to how best to manage the condition and prevent the development of severe complications such as acute renal failure and cerebral and pulmonary oedema are not available in the existing literature [25].

However, we describe here a case of a patient with restricting-type anorexia nervosa and concomitant rhabdomyolysis and hyponatremia, likely the consequence of excessive compulsive exercising, successfully managed by means of fluid restriction and solute repletion associated with an adapted inpatient version of an enhanced form of cognitive behavioural therapy (CBT-E).

## 2. Case Presentation 

We present the case of a 49-year-old female with restricting-type anorexia nervosa who was voluntary admitted for treatment at the Villa Garda Hospital Eating Disorder Unit, Italy, on 21 December 2015. The treatment based on an adapted inpatient version of CBT-E—an enhanced form of CBT designed to treat patients with eating disorder psychopathology—has a standard duration of 20 weeks, comprising 13 weeks of inpatient therapy followed by 7 weeks of day hospital [26, 27].

The patient reported no psychiatric disorders nor family history for eating disorders or any other significant psychiatric conditions. Her eating disorder had begun at the age of 30 years, when she had a body weight of 49 kg and a body mass index (BMI) of 19.6 kg/m^2^. After the death of her mother, the patient adopted a strict diet and excessive exercising (i.e., walking for long distances for a duration of 4-5 hours a day), which determined a progressive weight loss of about 10 kg over a few months and the onset of secondary amenorrhoea. In the years that followed the patient maintained a body weight of around 39 kg through extreme and rigid dietary rules and excessive daily exercising. At the age of 48 years, following the loss of her job and the breakup of a relationship, the patient stepped up both her dietary restriction and the duration of exercising and lost roughly 6 kg over the course of one year. At this point she was referred by her general practitioner to our eating disorder unit, which, after an initial assessment and consultation, she agreed to attend as an inpatient.

At admission the patient had a body weight of 33 kg, height of 1.57 m, and BMI of 13.4 kg/m^2^ as well as an oral temperature of 35.2°C, heart rate of 60 bpm, and blood pressure of 120/80 mm Hg, with no evidence of orthostatic changes. Her eating disorder psychopathology, assessed using the Italian version of the Eating Disorder Examination (EDE.170D) [28], was characterized by overvaluation of eating control, severe dietary restriction, and excessive exercising (about 4 to 5 hours of walking) so as to interfere with weight gain. The patient reported a persistent lack of recognition of the seriousness of her current low body weight and no episodes of binge eating or purging behaviours (i.e., self-induced vomiting and laxative or diuretic misuse) over the past three months. Her EDE global score was 1.83, which is slightly higher than 1 standard deviation (SD) above the community mean (i.e., above 1.74) [29].

Laboratory tests at admission, before refeeding commenced, showed marked reductions in natremia (123 mEq/L; normal range 136–144 mEq/L) and chloremia (89 mEq/L; normal range 101–111 mEq/L) and very high values of serum creatine kinase (3,238 IU/L; normal values: 38–234 IU/L). In association with the high serum creatine kinase levels, the patient also reported generalised skeletal pain, which was judged to be compatible with muscle injury and rhabdomyolysis due to excessive exercising.

At physical examination, the patient displayed no clinical signs of dehydration or distress (i.e., neurological, respiratory, cardiovascular, etc.) and neither her blood pressure nor her pulse rate increased when passing from a clinostatic to an orthostatic position. This, in association with her normal blood haemoglobin, hematocrit, albumin, total protein, urea nitrogen, and creatinine values and the absence of reported self-induced vomiting and laxative or diuretic misuse, was taken as indicative of nonhypovolemic hyponatremia [20]. Her urine specific gravity (1.011; normal range 1.005–1.030) was within the normal range and the serum osmolality (256 mOsm/kgH_2_O; normal range 275–295 mOsm/kg) was suggestive of the diagnosis of hypoosmolar hyponatremia [20].

The medical team judged the patient to be at a high risk of severe complications (i.e., cerebral and pulmonary oedema and acute renal failure) of hyponatremia and rhabdomyolysis secondary to excessive exercising, concluding that the best treatment option was inpatient CBT-E to address the patient's eating disorder psychopathology, including her excessive exercising, with some minor adaptations to treat the associated medical comorbidity.

In brief, inpatient CBT-E, described in detail elsewhere, has four main goals [19, 26]: (i) to engage the patient in treatment and change; (ii) to remove the eating disorder psychopathology; (iii) to correct the mechanisms responsible for maintaining the psychopathology; and (iv) to ensure the changes brought about are lasting. In this specific case, a major focus of the first part of the treatment was to help the patient to see the need to interrupt her excessive exercising, accept rest, and avoid movement, a strategy that would simultaneously manage her eating disorder psychopathology, rhabdomyolysis, and exercise-associated hyponatremia. To encourage the patient to make the decision to interrupt excessive exercising herself (rather than have it imposed on her), a fundamental principle of CBT-E, the patient was educated about the adverse effect of excessive exercising. We informed her that such behaviour was a potent maintenance mechanism of her eating disorder psychopathology [19] and current conditions, interfering with weight regain, likely underlying her muscle pain, rhabdomyolysis [30], and hyponatremia [5], and potentially causing overuse injuries, fractures, and adverse cardiac events, not to mention taking up time that could be used in other, more positive ways (e.g., doing enjoyable things with others).

As part of the engagement process, the patient was then involved in drawing up a personalized formulation including the main expressions of her eating disorder psychopathology (i.e., overvaluation of eating control, strict diet, excessive exercising, and low body weight) and the main mechanisms maintaining them. We then discussed with her the implications of the formulation on her treatment, the effects of her overvaluation of eating control, dietary restriction, and weight regain, and, in particular, the need for immediate interruption of exercising for skeletal healing and normalising exercise-associated hyponatremia. The patient was then assisted in creating a table outlining the “Pros and Cons of Change” and evaluating her reasons for and against excessive exercising. Since the patient remained reluctant to rest, we suggest that she try to stop exercising on a trial basis, which she voluntarily agreed to do, adopting a more collaborative approach. A similar procedure, described in detail in previous publications [26, 27], was used to help the patient to see her low weight as a problem and make the decision to address weight regain.

In order to manage her acute medical conditions, the patient was offered a two-week protocol that included the following strategies and procedures:

- Careful supervised water restriction (1.5 litres per day) to manage the euvolemic hyponatremia. The patient agreed to drink 500 mL water three times a day under a nurse's supervision and to refrain from drinking any more [31].
- Vitamin-B complex supplements to be prescribed for 21 days from the start of feeding to reduce the possibility of Wernicke's encephalopathy [32] as a consequence of refeeding syndrome and hyponatremia.
- Specific cognitive and behavioural procedures strategies to address the patient's excessive exercising, focusing on promoting rest, movement avoidance, and resisting the urge to exercise. These included real-time self-monitoring of the urge to exercise, considering the impulse to exercise as a tolerable temporary phenomenon and undertaking distracting activities with others. The patient was also encouraged to move only when necessary, in order to promote skeletal muscle healing and normalisation of the exercise-associated hyponatremia. Daily physical activity was monitored objectively by means of a SenseWear armband [33].
- A diet plan of 1500 kcal for the first week and then 2000 kcal in the second week, implemented as agreed upon with the patient, who had been informed that adequate caloric intake is highly recommended in muscle injury to promote healing and adequate solute repletion (sodium and protein) [34].
- Daily clinical examinations and regular laboratory tests (2-3 days), conducted during the first two weeks of feeding (Table 1) to monitor for refeeding syndrome and the potential onset of severe manifestations such as cerebral and/or pulmonary oedema and/or acute renal failure.

**Figure 1:**
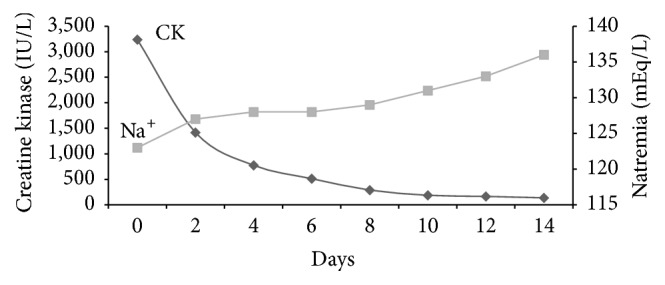
Decrease in creatine kinase and increase in natremia levels in the two-week period following inpatient admission.

The patient was compliant with the protocol, which brought about a rapid improvement in her clinical conditions. Indeed, after only 48 hours, CK levels had been reduced by almost 50% and these normalised within eight days (Figure 1). This prevented the occurrence of acute renal failure and was accompanied by the remission of muscle pain (Table 1). Laboratory tests also showed a gradual increase in serum sodium levels, which rose to normal levels in about 15 days (Figure 1), thereby preventing the onset of severe hyponatremia-related complications (Table 1). The patient, despite being extremely underweight, did not develop any signs of refeeding syndrome (Table 1).

Having completed the 13 weeks of inpatient therapy followed by 7 weeks of partial hospitalization, the patient was discharged on 11 May 2016 with a body weight of 48 kg, a BMI of 19.0 kg/m^2^, and a global EDE score of 0.48, which is less than 1 SD above the community mean (i.e., below 1.74) [29].

## 3. Discussion 

Rhabdomyolysis and hyponatremia have previously been reported separately in patients with anorexia nervosa as a consequence of excessive exercising [22–25] and purging behaviours [20], respectively. Although an association between hyponatremia and rhabdomyolysis has previously been reported in populations of athletes [2–4, 11], to our knowledge this is the first time that a case of both being induced by excessive exercising in a female patient with anorexia nervosa has been described.

In athletes, there are three main mechanisms involved in hyponatremia during exercise: (i) unequal replacement of salt and water lost in sweat (i.e., water replacement is greater than sodium); (ii) syndrome of inappropriate antidiuretic hormone release; and (iii) excess fluid intake (i.e., drinking a large amount of fluids over a short period of time) [2]. In our patient, the hyponatremia with corresponding hypoosmolality was suggestive of the syndrome of inappropriate antidiuretic hormone secretion (SIADH), likely determined by an increase in CK, as reported in athletes [7, 10]. It is also plausible that sodium depletion may have been further exacerbated by the prolonged malnutrition status [35] and loss through sweating. Indeed, the role of these mechanisms in producing hyponatremia is indirectly supported by correction of hyponatremia achieved via solute refeeding (sodium and proteins) and fluid restriction. Moreover, we would tend to exclude the role of excessive fluid intake in our patient, as she did not report it in her drinking history.

By understanding the underlying physiopathology and encouraging the active involvement of our patient, we were able to apply a well-timed protocol that rapidly resolved the existing medical manifestations of both and prevented the onset of severe life-threatening complications (e.g., acute renal failure and cerebral and pulmonary oedema).

This case report has several implications for clinicians working with patients with eating disorders. In particular, despite her severe clinical conditions, our patient was managed exclusively in a specialized inpatient unit for eating disorder. After a period of medical stabilization, this enabled the patient to seamlessly transition to the standard inpatient treatment for eating disorder, which led to complete weight restoration and improvement in her eating disorder psychopathology in the space of 20 weeks. Furthermore, this case highlights the need for clinicians to consider the effects of excessive exercising in patients with anorexia nervosa and to search for hyponatremia and rhabdomyolysis, even in the absence of purging behaviours [20] or psychogenic polydipsia [21]. It may be helpful in such cases to implement a protocol including cognitive behavioural strategies to encourage patients to get the rest they need and boost their calorie intake as well as monitoring of water intake and clinical and laboratory parameters to guard against the development of refeeding syndrome and/or the potentially life-threatening complications associated with hyponatremia and rhabdomyolysis.

## Figures and Tables

**Table 1:** Changes in clinical and laboratory variables in the two weeks following admission.

	0	2	4	6	8	10	12	14
Creatine kinase IU/L	3238	1417	775	513	289	187	165	138
Natremia (mEq/L)	123	127	128	128	129	131	133	136
Chloremia (mEq/L)	89	90	93	95	98	98	101	—
Creatinine (mg/dL)	0.53	—	0.52	—	0.55	—	0.56	—
Blood urea nitrogen (mg/dL)	19.09	—	11.06	—	11.57	—	10.36	—
Glycemia (mg/dL)	63	—	—	—	—	—	—	—
Phosphatemia (mg/dL)	2.6	2.8	2.5	2.9	3.5	3.4	3.3	3.7
Kalemia (mEq/L)	4.2	4.0	3.9	4.1	4.4	4.4	4.3	4.2
Magnesemia (mg/dL)	2.1	—	2.0	—	1.9	—	2.2	—
Body weight in kg	33	—	—	34	—	—	—	35
Caloric intake in kcal	1500	1500	1500	1500	2000	2000	2000	2000
Heart beats/minute	60	75	80	72	80	80	74	80
Daily steps per day^a^	1874	2179	2540	2732	2772	3610	4071	5795

^a^The daily steps per day were assessed by means of a SenseWear armband.
